# Supplementary material for: A new content-qualified antenatal care coverage indicator: Development and validation of a score using national health surveys in low- and middle-income countries
Source: J Glob Health. 2021 Jan 30;11:04008. doi: 10.7189/jogh.11.04008 (PMC7916319; doi:10.7189/jogh.11.04008)
Supplement: Online Supplementary Document [file jogh-11-04008-s001.pdf]

Table S1. Available variables in DHS and MICS surveys that could be used to construct the ANCq score. Source: DHS and MICS, 2010-2017.

Component	Question	Answer	Source DHS	MICS
**Cadre**	Did you see anyone for antenatal care for this pregnancy?	Y/N	X	X
	Whom did you see?	Doctor/nurse/midwife auxiliary/midwife/other person/traditional birth attendant community/ village health worker	X	X
**Where**	Where did you receive antenatal care for this pregnancy?	Your home/other home/govt. Hospital/govt. Health center/govt. Health if public or private post/post/another public sector/private med. Sector pvt. Hospital/clinic/Hospital/clinic/another private med. Sector/other	X	
**Gestational age at start**	How many months pregnant were you when you first received antenatal care for this pregnancy?	Months or DK	X	X
**Number of visits**	How many times did you receive antenatal care during this pregnancy?	Number of times or DK	X	X
**Blood pressure**	Was your blood pressure measured?	Y/N	X	X
**Urine sample**	Did you give a urine sample?	Y/N	X	X
**Blood sample**	Did you give a blood sample?	Y/N	X	X
**Weight**	Was your weight measured?	Y/N	X	X
**Height**	Was your height measured?	Y/N	X	X
**Ultrasound**	Did you have an ultrasound examination?	Y/N	X	X
**Risk**	During (any of) your antenatal care visit(s), were you told about things to look out for that might suggest problems with the pregnancy?	Y/N/DK	X	
**Tetanus**	During this pregnancy, were you given an injection in the arm to prevent the baby from getting tetanus, that is, convulsions after birth?	Y/N/DK	X	X
	During this pregnancy, how many times did you get a tetanus injection?	Number of times or DK	X	X
	At any time before this pregnancy, did you receive any tetanus injections?	Y/N/DK	X	X
	Before this pregnancy, how many times did you receive a tetanus injection?	Number of times or DK	X	X
	How many years ago did you receive the last tetanus injection before this pregnancy?	Years	X	X
**Iron**	During this pregnancy, were you given, or did you buy any iron tablets or iron syrup?	Y/N/DK	X	X
	During the whole pregnancy, for how many days did you take the tablets or syrup?	Days or DK	X	
**Worms**	During this pregnancy, did you take any drug for intestinal worms?	Y/N/DK	X	
**Malaria (not applicable to all countries)**	During this pregnancy, did you take any drugs to keep you from getting malaria?	Y/N/DK	X	X
	What drugs did you take?	Sulphadoxine Pyrimethamine /fansidar/chloroquine/other/DK	X	X
	How many times did you take (SP/Fansidar) during this pregnancy?	Number of times	X	X
	Did you get the (SP/Fansidar) during any antenatal care visit, during another visit to a health facility or from another	Antenatal visit / another facility visit /other source	X	

Component	Question	Answer	Source	
			DHS	MICS
	source?			

DHS: Demographic and Health Surveys; MICS: Multiple Indicator Cluster Surveys; Y: Yes; N: No; DK: Do not know.
X: indicates available data.

Table S2. Number of countries with available information on antenatal care variables. Source: DHS and MICS, 2010-2017.

Components	DHS (n=49)		MICS (n=50)		Total (n=99)	
	n	%	n	%	n	%
Whom did you see for antenatal care for this pregnancy?	47	96	50	100.0	97	98.0
Where did you receive antenatal care for this pregnancy?	46	94	3	6.0	49	49.5
How many months pregnant were you when you first received antenatal care for this pregnancy?	46	94	31	62.0	77	77.8
How many times did you receive antenatal care during this pregnancy?	47	96	50	100.0	97	98.0
As part of your antenatal care during this pregnancy was your blood pressure measured?	46	94	50	100.0	96	97.0
As part of your antenatal care during this pregnancy, did you give a urine sample?	46	94	50	100.0	96	97.0
As part of your antenatal care during this pregnancy, did you give a blood sample?	46	94	50	100.0	96	97.0
As part of your antenatal care during this pregnancy, was your weight measured?	18	37	8	16.0	26	26.3
As part of your antenatal care during this pregnancy, was your height measured?	7	14	3	6.0	10	10.1
As part of your antenatal care during this pregnancy, did you have an ultrasound examination?	2	4	8	16.0	10	10.1
During (any of) your antenatal care visit(s), were you told about things to look out for that might suggest problems with the pregnancy?	30	61	2	4.0	32	32.3
During this pregnancy, how many times did you get a tetanus injection?	41	84	35	70.0	76	76.8
Before this pregnancy, how many times did you receive a tetanus injection?	40	82	35	70.0	75	75.8
During this pregnancy, were you given, or did you buy any iron tablets or iron syrup? And how many days did you take the tablets or syrup?	45	92	3	6.0	48	48.5
During this pregnancy, did you take any drug for intestinal worms?	38	78	0	0.0	38	38.4
During this pregnancy, did you take any drugs to keep you from getting malaria?	24	49	14	28.0	38	38.4

DHS: Demographic and Health Surveys; MICS: Multiple Indicator Cluster Surveys.

Table S3. ANCq score distribution for each country by UNICEF region. Source: DHS and MICS, 2010-2017.

Country-year	Source	ANCq* – content qualified ANC score			
		Mean	Min-Max	Median	P25-P75
**West & Central Africa**					
Chad 2014	DHS	4.0	0 - 10	5	0 - 7
Niger 2012	DHS	4.8	0 - 10	5	4 - 7
Congo DR 2013	DHS	5.5	0 - 10	6	4 - 7
Mali 2015	MICS	5.9	1 - 10	6	4 - 7
Togo 2013	DHS	6.4	0 - 10	7	6 - 8
Burkina Faso 2010	DHS	6.6	0 - 10	7	6 - 8
Guinea 2016	MICS	7.0	0 - 10	7	6 - 8
Côte d'Ivoire 2016	MICS	7.2	2 - 10	7	6 - 8
Senegal 2017	DHS	7.4	0 - 10	8	7 - 9
Gabon 2012	DHS	7.9	0 - 10	8	7 - 9
Guinea Bissau 2014	MICS	7.5	3 - 10	8	7 - 8
Mauritania 2015	MICS	7.5	0 - 10	8	7 - 8
Benin 2014	MICS	7.3	1 - 10	7	6 - 9
Nigeria 2016	MICS	7.5	1 - 10	8	7 - 9
Gambia 2013	DHS	7.5	0 - 10	8	7 - 8
Congo 2014	MICS	7.9	1 - 10	8	7 - 9
Cameroon 2014	MICS	7.7	3 - 10	8	7 - 9
Liberia 2013	DHS	8.1	0 - 10	9	8 - 9
Ghana 2014	DHS	8.2	0 - 10	8	8 - 9
São Tome & Princ 2014	MICS	8.4	3 - 10	9	8 - 9
**Eastern & Southern Africa**					
Ethiopia 2016	DHS	4.2	0 - 10	5	0 - 7
Kenya 2014	DHS	5.2	0 - 10	5	4 - 7
Angola 2015	DHS	6.2	0 - 10	7	5 - 9
Burundi 2016	DHS	5.8	0 - 10	6	5 - 7
Zambia 2013	DHS	6.2	0 - 10	6	5 - 7
Tanzania 2015	DHS	6.4	0 - 10	7	5 - 8
Uganda 2016	DHS	6.5	0 - 10	7	6 - 8
Malawi 2015	DHS	6.4	0 - 10	7	6 - 7
Rwanda 2014	DHS	6.7	0 - 10	7	6 - 8
Comoros 2012	DHS	6.7	0 - 10	7	6 - 8
Zimbabwe 2015	DHS	6.9	0 - 10	7	6 - 8
Lesotho 2014	DHS	7.4	0 - 10	8	7 - 9
South Africa 2016	DHS	7.3	0 - 10	8	7 - 8
Namibia 2013	DHS	7.5	0 - 10	8	7 - 9
Eswatini 2014	MICS	7.9	4 - 10	8	7 - 9
**Middle East & North Africa**					
Yemen 2013	DHS	4.0	0 - 10	5	0 - 7
Egypt 2014	DHS	6.5	0 - 10	7	5 - 9
Sudan 2014	MICS	7.2	1 - 10	7	6 - 8
Jordan 2017	DHS	8.4	0 - 10	9	8 - 9
**South Asia**					
Afghanistan 2015	DHS	3.5	0 - 10	4	0 - 6
Pakistan 2017	DHS	6.5	0 - 10	7	5 - 9

Country-year	Source	ANCq* – content qualified ANC score			
		Mean	Min-Max	Median	P25-P75
India 2015	DHS	6.8	0 - 10	8	6 - 9
Nepal 2016	DHS	6.9	0 - 10	8	6 - 9
Maldives 2016	DHS	9.1	0 - 10	9	9 - 10
**East Asia & the Pacific**					
Myanmar 2015	DHS	6.2	0 - 10	7	5 - 8
Timor Leste 2016	DHS	6.6	0 - 10	7	6 - 9
Indonesia 2012	DHS	7.4	0 - 10	8	7 - 9
Cambodia 2014	DHS	7.3	0 - 10	8	7 - 9
Philippines 2017	DHS	7.8	0 - 10	8	7 - 9
Vietnam 2013	MICS	7.8	2 - 10	8	7 - 9
Thailand 2015	MICS	8.9	4 - 10	9	8 - 10
**Latin America & Caribbean**					
Haiti 2016	DHS	7.3	0 - 10	8	7 - 9
Guatemala 2014	DHS	7.6	0 - 10	8	7 - 9
Honduras 2011	DHS	8.0	0 - 10	8	8 - 9
Guyana 2014	MICS	8.0	1 - 10	8	7 - 9
Colombia 2015	DHS	8.5	0 - 10	9	8 - 10
Belize 2015	MICS	8.7	5 - 10	9	8 - 9
El Salvador 2014	MICS	8.6	3 - 10	9	8 - 9
Mexico 2015	MICS	8.7	2 - 10	9	8 - 10
Peru 2016	DHS	8.9	0 - 10	9	8 - 10
Paraguay 2016	MICS	9.1	2 - 10	9	9 - 10
Dominican Rep 2014	MICS	9.3	3 - 10	10	9 - 10
Cuba 2014	MICS	9.3	7 - 10	9	9 - 10

DHS: Demographic and Health Surveys; MICS: Multiple Indicator Cluster Surveys.
Min-Max: minimum and maximum values; P25- P75: 25th and 75th percentiles.
*The theoretical ANCq score range is between 0 and 10.

Table S4. Studies measuring antenatal care quality in multicountry analyses.

Author/Paper	Objective	Variables considered to measure ANC quality	ANC quality measurement	Included women without ANC	Source of information	Number of surveys included in each paper	Number of surveys included in the AUC analyses
**Arsenault et al. (2018)** Equity in antenatal care quality: an analysis of 91 national household surveys.	To describe inequalities in antenatal care quality across the largest possible set of countries using comparable indicators and a standardized measurement approach	Blood pressure monitoring<br>Urine testing<br>Blood testing	Antenatal care quality was included as a binary outcome measuring the proportion of women who received the three interventions at any point during pregnancy among those who sought care from skilled providers	No	DHS and MICS	91 LMICs	63
**Carvajal-Aguirre et al. (2017)** Gap between contact and content in maternal and newborn care: An analysis of data from 20 countries in sub-Saharan Africa.	To describe the gap between contact and content –as a proxy for quality– of maternal and newborn health services by assessing level of co– coverage of antenatal care and postnatal care interventions	Urine test<br>Blood pressure taken<br>Blood sample<br>Iron supplementation<br>Tetanus protection<br>Counselled on pregnancy complications<br>Tested for HIV and received results<br>Intermittent preventive treatment of malaria in pregnancy	Proportion of women with at least one ANC visit and those with four or more visits who received all 8 interventions	No	DHS	20 sub–Saharan Africa countries	14

Author/Paper	Objective	Variables considered to measure ANC quality	ANC quality measurement	Included women without ANC	Source of information	Number of surveys included in each paper	Number of surveys included in the AUC analyses
**Amouzou et al. (2017)** Skilled attendant at birth and newborn survival in Sub-Saharan Africa.	To reassess the relationship between skilled attendant at birth and neonatal mortality by distinguishing deaths on the first day of life from those on days 2–27. Also, to assess the association between the receipt of a series of seven antenatal care and two immediate postnatal care interventions by women and their newborns and mortality on days 0–1 and days 2–27	Urine test<br>Blood test<br>Blood pressure measured<br>Iron supplementation<br>Tetanus protection at birth<br>Counselled on pregnancy complications<br>Tested for HIV and received results	Was created a categorical variable of number of interventions received by summing the indicator variable representing each intervention	Yes	DHS	20 sub–Saharan Africa countries	23

ANC: Antenatal care; AUC: Area under the ROC curve; DHS: Demographic and Health Surveys; MICS: Multiple Indicator Cluster Surveys; LMICs: Low and middle-income countries

Table S5. Additional variables and number of surveys with available information included in the extended principal component analysis (PCA).

PCA	Additional variables	Number of surveys	N
ANCq	No additional variables	63	583,602
Extended1	Iron supplementation for 90 days or more, information about pregnancy complications	27	399,631
Extended2	Iron supplementation for 90 days or more, information about pregnancy complications, HIV tested and HIV results	23	338,942
Extended3	Iron supplementation for 90 days or more, information about pregnancy complications, weight, and height measurement	5	45,009
Extended1	Iron supplementation for 90 days or more, information about pregnancy complications, weight, and height measurement, HIV tested and HIV results	4	29,888

PCA: principal component analysis; HIV: human immunodeficiency virus.

Table S6. Correlation coefficients and extended principal component analysis (PCA) association with neonatal mortality. Source: DHS, 2010-2017.

PCA	N countries	Correlation with ANCq		Extended		ANCq*	
		Spearman	Pearson	OR	95% CI	OR	95% CI
Extended1	27	0.95	0.99	0.87	(0.86, 0.88)	0.87	(0.86, 0.88)
Extended2	23	0.93	0.98	0.87	(0.86, 0.88)	0.87	(0.86, 0.88)
Extended3	5	0.93	0.96	0.84	(0.81, 0.88)	0.81	(0.76, 0.86)
Extended4	4	0.89	0.95	0.88	(0.84, 0.92)	0.84	(0.79, 0.90)

PCA: principal component analysis; DHS: Demographic and Health Surveys; OR: Odds Ratio; CI: confidence interval.
*Logistic regression models included only the countries with all variables used in the extended set of variables

Figure S1. Forest plot showing the country-level association of ANCq with neonatal mortality, plus the pooled crude odds ratio (based on a random effect model), using 42 DHS. Source: DHS, 2010-2017.

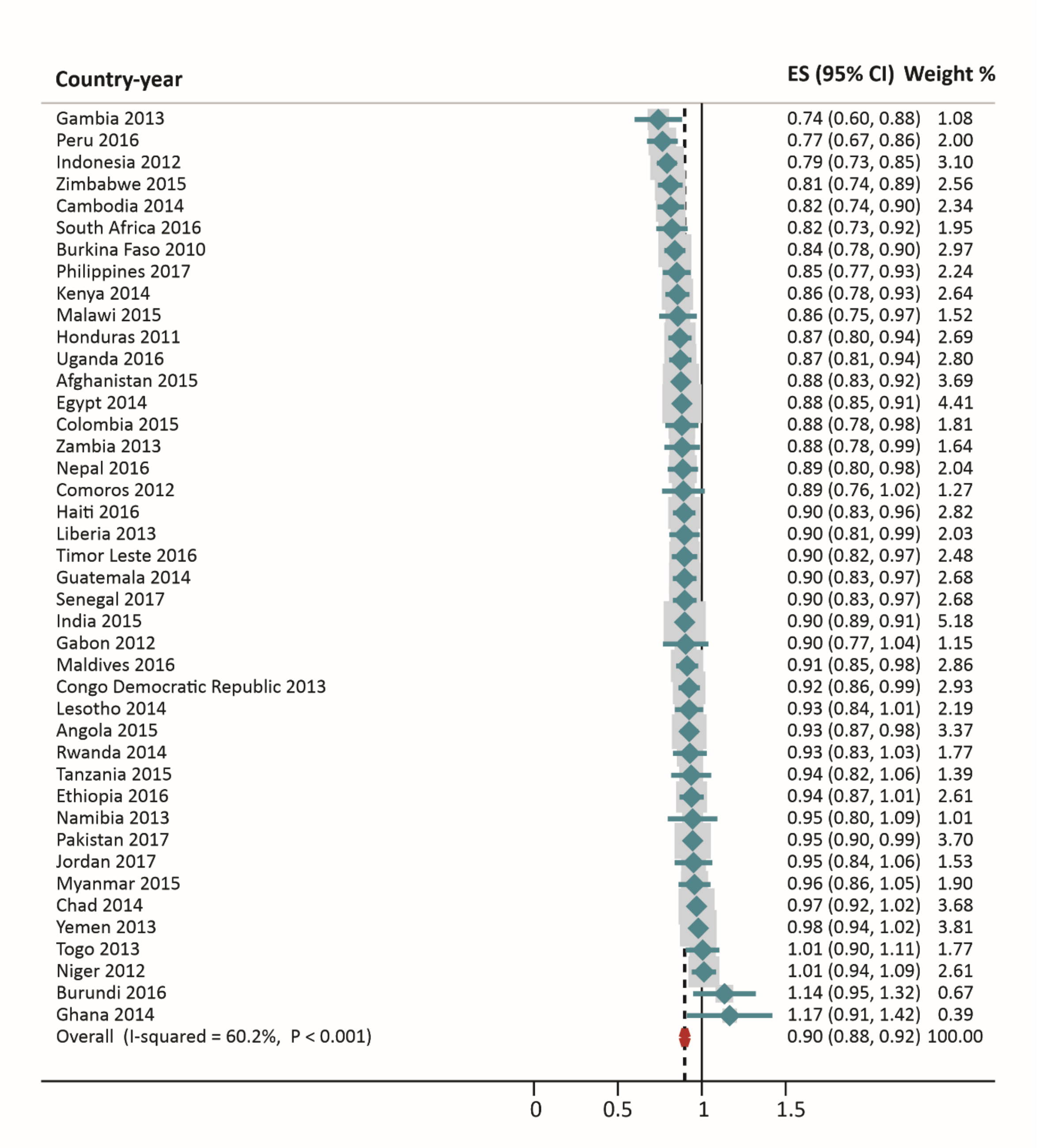

DHS: Demographic and Health Surveys; ES: effect size; CI: confidence interval.
The dotted red line represents the pooled effect size.
